# The Disorders of Digestion in Infancy and Childhood

**Published:** 1897-10

**Authors:** 


THE DISORDERS OF DIGESTION IN INFANCY AND CHILDHOOD. By W. SOLTAU FENWICK, M. D., B. S., London, Member of the Royal College of Physicians, etc. Illustrated. Philadelphia: J. B. Lippincott Company. 1897.

The author has based his conclusions as presented in this book chiefly on his experience in the Evelina Hospital, where some 5,000 cases of digestive disturbance have passed under his observation.

Considerable investigation has been undertaken by the author, the most interesting of which is his work in the analysis of the stomach contents in various disorders of digestion in infancy. A number of original drawings and microphotographs illustrate his findings in morbid anatomy. While some work in the bacteriology of summer diarrhea was done by the author, which he supplements by quoting from others, the more recent and thorough research in this field by Booker makes the small space devoted to this of little value.

In the matter of treatment the book is in the main satisfactory, though not so in regard to the diet of infancy. Those familiar with the work of Rotch, Holt and others will not read the chapter on dietetics a second time. The author himself acknowledges the more progressive attitude of American and continental pediatrists in this respect.

The book is not as complete in some features as one feels that the author's experience might have made it, yet it contains much which the practitioner will find helpful and suggestive.

